# Cell Wall Polysaccharide Composition of Grafted ‘Liberty’ Watermelon With Reduced Incidence of Hollow Heart Defect

**DOI:** 10.3389/fpls.2021.623723

**Published:** 2021-03-04

**Authors:** Marlee A. Trandel, Suzanne Johanningsmeier, Jonathan Schultheis, Chris Gunter, Penelope Perkins-Veazie

**Affiliations:** ^1^Postharvest Laboratory, Department of Horticultural Sciences, Plants for Human Health Institute, North Carolina State University, Kannapolis, Kannapolis, NC, United States; ^2^United States Department of Agriculture – Agricultural Research Service (USDA-ARS), Food Science Market Quality and Handling Research Unit, Department of Food, Bioprocessing and Nutrition Sciences, North Carolina State University, Raleigh, Raleigh, NC, United States; ^3^Vegetable Extension, Department of Horticultural Sciences, North Carolina State University, Raleigh, Raleigh, NC, United States

**Keywords:** *Citrullus lanatus*, watermelon, neutral sugar, graft, linkage assembly, pectin

## Abstract

Grafting watermelon scions to interspecific squash hybrids has been found to increase fruit firmness. Triploid (seedless) watermelon are prone to hollow heart (HH), an internal fruit disorder characterized by a crack in the placental tissue expanding to a cavity. Although watermelon with lower tissue firmness tend to have a higher HH incidence, associated differences in cell wall polysaccharide composition are unknown. Grafting “Liberty” watermelon to “Carnivor” (interspecific hybrid rootstock, *C. moschata* × *C. maxima)* reduced HH 39% and increased tissue firmness by 3 N. Fruit with and without severe HH from both grafted and non-grafted plants were analyzed to determine differences in cell wall polysaccharides associated with grafting and HH. Alcohol insoluble residues (AIR) were sequentially extracted from placental tissue to yield water soluble (WSF), carbonate soluble (CSF), alkali soluble (ASF), or unextractable (UNX) pectic fractions. The CSF was lower in fruit with HH (24.5%) compared to those without HH (27.1%). AIRs were also reduced, hydrolyzed, and acetylated for GC-MS analysis of monosaccharide composition, and a portion of each AIR was methylated prior to hydrolysis and acetylation to produce partially methylated alditol acetates for polysaccharide linkage assembly. No differences in degree of methylation or galacturonic and glucuronic acid concentrations were found. Glucose and galactose were in highest abundance at 75.9 and 82.4 μg⋅mg^–1^ AIR, respectively, followed by xylose and arabinose (29.3 and 22.0 μg⋅mg^–1^). Mannose was higher in fruit with HH (*p* < 0.05) and xylose was highest in fruit from grafted plants (*p* < 0.05). Mannose is primarily found in heteromannan and rhamnogalacturonan I side chains, while xylose is found in xylogalacturonan or heteroxylan. In watermelon, 34 carbohydrate linkages were identified with galactose, glucose, and arabinose linkages in highest abundance. This represents the most comprehensive polysaccharide linkage analysis to date for watermelon, including the identification of several new linkages. However, total pectin and cell wall composition data could not explain the increased tissue firmness observed in fruit from grafted plants. Nonetheless, grafting onto the interspecific hybrid rootstock decreased the incidence of HH and can be a useful method for growers using HH susceptible cultivars.

## Introduction

Seedless (triploid) watermelon now make up 90–95% of the U.S. market (Levi et al., 2014) and are prone to an internal fruit disorder known as hollow heart (HH), characterized by a cavity or void air space in the center of the fruit (Johnson, 2014, 2015). Triploid watermelon do not produce viable pollen, thereby requiring diploid (seeded) pollenizer plants at correct ratios (25–33% of field) to avoid the onset of HH (Freeman et al., 2007). Inadequate pollination from reduced bee visits or limited pollen release due to cold or wet weather also causes HH in watermelon (Johnson, 2015; Trandel et al., 2020a). HH disorder is thought to develop at the epidermal layers within the carpels, ovule and septum (mesocarp or placental tissue) (Kano, 1993). Watermelon rind is known to expand and differentiate for the duration of fruit growth. In contrast, mesocarp tissue division stops 7–10 days after fruit set, tissue growth slows and instead the placental cells enlarge with water, sugars, proteins and nutrients. If the relative rates of rind and mesocarp growth are too far apart, the three internal fruit compartments will separate, leading to HH (Kano, 1993). This internal fruit disorder has been associated with textural changes including decreased tissue firmness and cell density (e.g., increased cell size) (Kano, 1993). In a multi-year, large cultivar study of triploid selections, cultivars that produced fruit with lower tissue firmness had a higher incidence of HH (Trandel et al., 2020a). However, grafting of triploid watermelon onto squash rootstock shows promise for both increasing watermelon flesh firmness and reducing the incidence of HH disorder (Trandel et al., 2020b).

Grafting watermelon onto *Cucurbita* rootstocks (RS) has been used as a means to reduce damage caused by soil-borne diseases (Mohamed et al., 2012). Grafting watermelon to interspecific hybrid RS (*Cucurbita maxima* × *C. moschata*) has been reported to change fruit textural characteristics including increased tissue firmness (Soteriou et al., 2014, 2017) and possibly increased cell density (e.g., smaller cells and more cells/unit area). Furthermore, Soteriou et al. (2017) found a positive correlation between tissue firmness of fruits from non-grafted plants or those grafted onto interspecific hybrid RS and the sum of insoluble pectic fractions (%CSF + %ASF). This suggests that differences in watermelon textural characteristics may be from de-esterified pectins and matrix glycans (Brummell and Harpster, 2001). Since fruit firmness has been associated with incidence of HH (Trandel et al., 2020a) and differences in pectic substances, a closer look into the cell wall components of watermelon fruit is warranted.

A primary cell wall is ubiquitous among all plant tissues and serves a variety of functions, including cell wall strength, integrity, and protection of intracellular contents (Rose, 2003; Brummell, 2006). The cell wall maintains fruit structural integrity as cell size expands and associated extracellular air spaces develop during fruit growth and maturation (Ng et al., 2013). In general, plant cell walls are composed of polysaccharides (90% of dry weight), structural glycoproteins (2–5%), phenolic esters (<2%), and minerals (5%) (Rose, 2003). Cell wall polysaccharides commonly include cellulose, hemicellulose (matrix glycans) and pectin. During fruit growth and development, the cell wall undergoes protoplast-controlled modification in both morphology and composition (Tanner and Loewus, 2012). Pectic polysaccharides are deposited during the formation of new cell walls in the process of cell plate formation (Amos and Mohnen, 2019). The plate persists during deposition of the primary cell wall and layers of pectic polysaccharides are secreted then pushed outward via internal cell pressure (Tanner and Loewus, 2012), resulting in a pectin-rich middle lamella (Amos and Mohnen, 2019). Crosslinking of pectin within the middle lamella strongly controls intercellular junction and cellular adhesion (Ng et al., 2013; Daher and Braybrook, 2015; Amos and Mohnen, 2019). Pectin depolymerization, de-esterification (e.g., breakdown of pectin), and changes in cross-linkage within the middle lamella occur during fruit ripening, causing loss of cell-to-cell adhesion (Ng et al., 2013; Amos and Mohnen, 2019). Pectin breakdown can affect cell wall strength (Daher and Braybrook, 2015) and contributes to loss of tissue firmness and diminishing flesh quality (Brummell, 2006; Amos and Mohnen, 2019).

Ripe watermelon fruit have a crisp and juicy texture rather than the soft and melting flesh texture associated with many tree fruits and berries (Harker et al., 1997). The fruit undergoes changes in pigment, flavor, soluble solids, and tissue firmness during ripening. As the fruit matures, photosynthesis, and nutrient uptake increases and intercellular spaces fill with sugars and water (Guo et al., 2011). At maturity, the tissue consists of very large cells encompassed by a primary cell wall and separated by intercellular spaces. Once overripe, the flesh turns bright red with accumulation of volatile compounds, tissue firmness, and placental tissue becomes dry with seed trace cavities apparent (Guo et al., 2011).

Polysaccharide changes were followed in watermelon treated with ethylene to stimulate polygalacturonase, depolymerize pectic substances in the cell wall and soften fruit texture (Karakurt et al., 2008; Mort et al., 2008). Despite changes in fruit firmness, no change in monosaccharide composition or matrix glycans were found (Karakurt et al., 2008; Mort et al., 2008). Differences in xyloglucan/xylose monomers and enzymes known to have hydrolytic activity were also followed (Karakurt et al., 2008; Mort et al., 2008; Karakurt and Huber, 2009). First watermelon cell walls were hydrolyzed with endo-polygalacturonase and assessed using NMR via hydrogen and carbon shifts (^1^H and ^13^C) shifts (Karakurt et al., 2008). Nine linkage residues were identified consisting of xylose [1-Xyl(p)] and galacturonic acid [3-GalA(p), 4-GalA(p), and 3,4-GalA(p)] linkages, but no differences were seen in monomers or linkage residues related to ethylene induced tissue softening (Mort et al., 2008). Then, enzymes from xylosyl linked residues were analyzed in watermelon treated with and without ethylene induced water soaking (Karakurt and Huber, 2009). Five endo-xyloglycan transferase enzymes (P1S1, P2S2, P3S1, P3S2, and P3S3) were found in watermelon and no differences were found with and without an ethylene treatment. The enzymes were also active toward carboxymethylcellulose, indicating they were not specific to xylose/xyloglucan and did not play a dominate role in tissue softening from ethylene induced watermelon (Karakurt and Huber, 2009).

Watermelon grafted to squash rootstocks showed differential expression in genes associated with primary and secondary metabolism (Liu et al., 2017; Soteriou et al., 2017), including carbohydrate metabolism. These subsequent gene changes from grafting may either increase or decrease cell wall monomers/linkage residues. Information is available on the cell wall polysaccharide composition of cucurbit species that could be used as rootstocks. Total pectin, molecular weight, and monosaccharide analysis was previously studied on microwave heated/pH extracted *Cucurbita maxima* cell walls (Yoo et al., 2012) and to identify differences of *Cucurbita maxima* vs. *Cucurbita pepo* Lady Godiva (Song et al., 2011; Zhou et al., 2014). Exposing cell walls to lower pH (pH 1.0–1.5) increased total pectic yields while microwave temperature had no effect (Yoo et al., 2012). Zhou et al. (2014) elucidated polysaccharide linkage in *Cucurbita maxima* and found high amounts of galactose residues (2,3,4,6-Gal, 2,3,6-Gal and 2,4-Gal) in pumpkin. In Lady Godiva pumpkins (*Cucurbita pepo*) crude polysaccharides were extracted and molecular weight, monosaccharides and chemical structures were identified (Song et al., 2011). Glucose (50%), galactose (41.67%), and fucose (8.33%) were monomers in highest composition and galactose (2,3,4-Galp, 3,4-Galp), glucose (2,3,4,6-Glcp, 2,4,6-Glcp, 2,3-Glcp) and terminal fucose linkages (2,3,4-fucp) were identified via methylation analysis (Song et al., 2011). In *Cucurbita pepo* higher amounts of fucose were identified in comparison to watermelon (Song et al., 2011), while galactose and glucose were the most abundant monomers in both pumpkin and watermelon (Karakurt et al., 2008; Song et al., 2011). Therefore, grafting watermelon to interspecific RS may increase the amount of fucose and highly branched glucose and galactose linkage residues, which play a critical role in rhamnogalacturonan II (RG II) branching (Brummell and Harpster, 2001). Researchers propose that crosslinking of homogalacturonan (HG) and RG II within the middle lamella strongly controls cellular adhesion and differences in fruit textural characteristics (Daher and Braybrook, 2015; Amos and Mohnen, 2019). In the present study, we hypothesize that grafting watermelon to interspecific rootstocks will increase tissue firmness and reduce the incidence of HH by increasing total pectin content, monomeric building blocks and glucose and galactose linkage residues ultimately increasing cell wall communication, strength, and integrity.

The objective of this study was to explore differences in watermelon cell wall polysaccharide composition relative to HH and grafting, using fruit from a large field study where HH was induced via reduced pollinezer plants. Cell wall polysaccharide characterization was done through assessment of total pectin content, and quantification of neutral sugars and uronic acids of pectic fractions. Total pectin content and sequential fractions of water soluble (WSF), carbonate soluble (CSF), alkali soluble (ASF) and unextractable fractions have been studied and are known to contain specific polysaccharide types (Brummell and Harpster, 2001; Paniagua et al., 2014; Soteriou et al., 2017). Determination of neutral sugar composition and linkage assembly was conducted to identify differences in cell wall polysaccharide composition with the onset of HH or grafting. Determination of cell wall polysaccharide composition, such as cellulose, hemicellulose and pectin, relied on assays to deduce the various components through identification of monosaccharide composition and associated linkage assembly (Pettolino et al., 2012). Saemen hydrolysis was used on intact cell wall samples to cleave polysaccharides into monomeric building blocks (Saeman et al., 1954) and was used to quantitatively measure 12 neutral monosaccharide building blocks (Pettolino et al., 2012). Complete methylation of the cell wall was used to deduce linkage residues and estimate the amount of polysaccharide classes. Understanding the composition of the cell wall with these methods could lead to a better understanding of general fruit textural changes with grafting and may be useful in elucidating how HH forms in watermelon.

## Materials and Methods

### Plant Material

Triploid and diploid watermelon were grown as transplants. “Liberty” triploid watermelon (Nunhems USA Inc., Parma ID) was used as the scion and not grafted or grafted onto the interspecific hybrid RS (*Cucurbita maxima* × *C. moschata*) “Carnivor” (Syngenta AG, Basal Switzerland). “Liberty” seed was sown on 29 April 2019, 7 days prior to “Carnivor.” The diploid pollinezer “SP-7” (Syngenta AG, Basal Switzerland) was sown on 16 April 2019. A one-cotyledon graft method was utilized on 11 May 2019 following the method of Hassell et al. (2008). Grafted plants were placed into a healing chamber at a constant 27°C, 100% humidity provided by a Trion Comfortbreeze CB777 Atomizing Hurmidifier (Trion, Sanford NC). Following a 6 days graft union healing interval, the plants were held in an open wall greenhouse to harden off for 2–3 days.

The experiment was carried out via open field production and was planted at the Cunningham Research Center (35.8942 °N, 77.6801 °W) in Kinston, NC from May to August 2019. Six watermelon beds (rows) were used and were split evenly into two blocks via a 7 m drive row. The experimental design was a two factor, full factorial design with grafting treatments and HH phenotype as the main effects.

The study field was fumigated with Telone II (Dow AgroSciences, Indianapolis, IN) via broadcast application at 65.6 kg⋅ha^–1^ on 30 April 2019. S-Metachlor (1.7 kg⋅ha^–1^), Terbacil (0.2 kg⋅ha^–1^), and Glyphosate (2.3 kg⋅ha^–1^) (Dow AgroSciences) were applied between plastic beds for weed control in April before planting.

**FIGURE 1:**
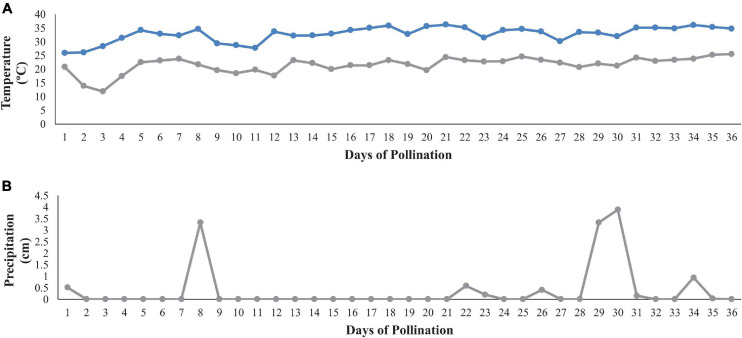
Maximum and minimum daily temperatures **(A)** and daily precipitation **(B)** during the estimated time of fruit set (3–7 weeks after transplanting). The x-axis represents the estimated number of days of pollination with diploid flowers opening ∼ 13 June 2019; climate data shown from 13 June to 18 July.

Preplant broadcast fertilizer 10-10-20 (10.0N-4.35P-16.5K) (Helena 10-10-20 Broadcast Fertilizer, B.B. Hobbs, Clinton, NC) was applied on 24 April 2019 at 67.3 kg⋅ha^–1^ N, 67.3 kg⋅ha^–1^ P and 134.5 kg⋅ha^–1^ K, respectively. Watermelon were planted on 22 May 2019 and one block (3 rows) was planted with non-grafted plants and the second block (3 rows) was planted with grafted transplants. Rows were 100 m long, with 3.1 m between row spacing and 0.8 m in-row spacing for graft and non-graft watermelon plants. To induce HH, the diploid pollenizer “SP-7” was transplanted every 12 m in each of the rows and held at 8% of the total plant population. Typically, growers plant between 25 and 33% of the production field is panted in diploid plants for adequate pollination and fruit set. Trickle irrigation (NETAFIM, 197 ml, 0.24 gph; NETAFIM, Tel Aviv, Israel) was utilized over the course of the growing season. Liquid fertigation started 2 weeks after planting and was applied weekly using a 7-0-7 (7.0N-0.0P-5.8K) liquid fertilizer (Liberty Fertilizer, B.B. Hobbs, Clinton NC). Cumulative amounts of fertilizer (broadcast and drip) applied over the growing season were 95.3 kg⋅ha^–1^ N, 67.3 kg⋅ha^–1^ P, and 162.5 kg⋅ha^–1^ K, respectively. A conventional spray program from North Carolina was used for the duration of production (Schultheis and Starke, 2019). Watermelon harvest began 30 July 2019 and ended 06 August 2019 (71 and 77 days from transplant). The maximum and minimum daily temperatures and daily precipitation during pollination and fruit set (3–7 weeks after transplant) are reported in Figure 1.

### Quality Evaluations

Fruit were weighed and cut longitudinally from stem to blossom end then rated for the incidence and severity of HH with a 1–5 scale (1 = no HH to minor crack and 5 = severe cavity) following USDA (2006) grading standards. Fruit were subjectively rated for ripeness based on color development and tissue breakdown in the seed trace cavity areas. One fruit half was saved for cell wall polysaccharide and total pectin sample extraction. Rind firmness and tissue firmness (N) were assessed as maximum resistance to puncture (5 mm depth) at two locations near or in heart tissue using a stationary firmness tester equipped with a Force One FDIX Digital Force Gauge (Wagner Instruments, Greenwich, CT) and a 0.8 cm diameter flat tip probe (Perkins-Veazie et al., 2016). Following flesh firmness readings, the fruit half was flipped over and rind firmness was determined using a needle style probe (0.3 cm diameter) that penetrated both the rind and peel. Total number of grafted fruit samples was 102 and 19 had HH. Total number of non-grafted fruit samples was 138 and 73 had HH.

A central core of tissue (∼5 g) was cut from the half, squeezed onto a digital refractometer (Atago Pal-1, Bellevue WA) and soluble solids (°Brix) content determined. A second set of tissue samples (100 g) were taken from the heart area, frozen, thawed, and pureed for determination of pH, soluble sugars (sucrose, fructose, and glucose), free citrulline, free arginine and total lycopene following the methods of Fall et al. (2019).

### Mineral Analysis on Watermelon Flesh Tissue

A sample from the central core (∼50 g) of the watermelon half used for tissue firmness determination was excised for mineral analysis. In cases of severe HH, the sample was taken directly from the hollowed area. The sample was checked for seed cavities/seed traces and if any were present, they were removed. Sample weight was recorded, and samples were frozen in disposable tubes −80°C for 1 day then freeze dried for 10 days. Freeze dried samples were analyzed for mineral content (A&L Great Lakes Laboratory, Fort Wayne, IN) as described below.

Samples were dried overnight at 100–105°C then ground with a Wiley Mill Grinder and sieved through a 20 mesh screen. Total nitrogen was measured via the Dumas Method (Saint-Denis and Goupy, 2004) and nitrate and nitrite were measured by cadmium reduction and colorimetric analysis by flow injection system (Yue et al., 2004). Sample digestion for mineral analysis was accomplished using hot acid extraction via a vessel microwave procedure. Samples were weighed to ∼0.2 g, 2 mL of nitric acid added and samples microwaved in an oven programmed to ramp up to 90°C and held for 90 s. Samples were cooled below 50°C and 1 mL of peroxide was added, then returned to the microwave oven and ramped up to 105°C and held for 10 min. After the samples cooled, the samples were brought to a final volume of 25 mL (∼1:125 dilution), capped, mixed and minerals were analyzed via Inductively Coupled Argon Plasma with a multispectral detector.

### Experimental Design to Analyze Cell Wall Polysaccharides

Cell wall analysis was set up as a randomized complete block design (RCBD) to assess the cell wall architecture of watermelon fruits with and without HH from grafted and non-grafted plants. Samples with or without severe HH and of similar ripeness as determined by SSC and pH were selected for cell wall analysis. Six fruit were analyzed for each phenotypic group. A total of three batches (24 fruit samples) were analyzed for total pectin and cell wall polysaccharides. Samples were blocked by analysis batch. Each batch was comprised of a randomized sampling of watermelon AIRs that included two watermelon fruit for each of the four treatment combinations per batch.

### Total Pectin

Samples for total pectin content (50 g) were cut with a sharp knife from the center portion of each watermelon heart, and frozen at −80°C. About 20 fruit per graft and HH combination were used to collect samples. Samples with no HH and severe HH ratings (4–5) were selected for analysis. About 20 g of frozen tissue were weighed into 50 mL disposable test tubes for total pectin analysis. Samples were ground in 80% ethanol and further extracted with acetone and methanol to yield AIR. Total pectin methods follow Trandel et al. (2020b).

Yields of AIR were ∼40–50 mg and were then sequentially extracted with ultrapure water, 50 mM calcium carbonate and 4 M potassium hydroxide to create water-soluble (WSF), carbonate soluble (CSF), and alkali soluble pectic fractions (ASF). The cell wall material was filtered, and filtrates were saved between sequential extractions. The sequential fraction filtrates were freeze dried and the weight of the fraction was obtained to calculate the percentage of each fraction relative to the starting AIR. The freeze-dried fractions were diluted with 20 mL of di-water and total neutral sugars (Masuko et al., 2005) and uronic acids (Blumenkrantz and Asboe-Hansen, 1973) were determined on all sequential fractions. Galactose was used as the calibration standard for total neutral sugars and galacturonic acid was used as the calibration standard for total uronic acids. Results were expressed in galacturonic acid and/or galactose equivalents per g of cell wall material from each of the sequential extractions.

### Sugar Standards for Cell Wall Polysaccharide Composition Analysis

Twelve sugar standards (meso-erythritol, 2-deoxy-d-ribose, rhamnose, fucose, ribose, arabinose, xylose, 2-deoxy-d-glucose, allose, mannose, galactose, and glucose) and the internal standard sugar of myo-inositol were used for cell wall quantification. The standard sugars were dried in a vacuum (Isotemp Vacuum oven, Model 2855, Fisher Scientific) oven at 40°C overnight in the presence of desiccant (added to the oven in an open metal tray to collect water from the sugar standards). Sugar standards (0.1 M) were prepared from the dry sugars and double distilled water then used to prepare a stock mix of the 12 sugars. The stock sugar mix was diluted at 1:5, 1:10, 1:15, and 1:20 for the calibration curve. Myo-inositol was diluted at 1:20. The internal standard and stock sugar mixes were stored at −80°C until ready for use.

### Cell Wall Polysaccharide Extraction

Methods for cell wall analysis were adapted from Pettolino et al. (2012). Details on optimization of cell wall polysaccharide extraction on watermelon can be found in Trandel et al. (2020b). In brief, ∼10 g of heart tissue was excised, frozen with liquid N and packed into 50 ml test tubes then stored at −80°C. Each of the frozen watermelon samples were ground in 80% ethanol similar to total pectin methods and washed with solvents to yield AIR. AIRs were placed in a fan forced oven for 16 h at 37°C to dry.

Prior to starting the reduction steps, the dried AIRs were packed into 2 mL microcentrifuge tubes (PFSS 2800 50 20U, OPS Diagnostics, Lebanon NJ) and re-ground with 4 × 2.8 mm steel balls (magnetic, high carbon, Grainger, Lake Forest, IL) for 60 s at 4,000 strokes/min using a bead mill (BeadBug Microtube Homogenizer, Model D1030, Sigma Aldrich, St. Louis, MO). Particle size was analyzed via Helos laser diffraction particle analyzer (Sympatec, Clausthal-Zellerfeld, Germany). The final particle size of the ground AIRs yielded 55.3 ± 0.43 μm. Samples went through a series of carboxyl reductions (reduction I and II). Small molecules were filtered, and large molecules were recovered (<3 KDa) using centrifugal ultrafiltration (Vivaspin turbo 15 mL Centrifugal Ultrafiltration Filters; Sigma Aldrich, Germany, Darmstadt). Samples were quantitatively transferred to the Ultrafiltration centrifugal filters. The glass test tube was rinsed 3–4 times with 500 μL of di-water. Then samples were centrifuged (Sorval Legend RT Centrifuge, Model D-37520 Osterode) at 4,000 *g* for 1.5 h (or until ∼200 μL of retentate remained). Following the second reduction, samples were quantitatively split for hydrolysis or methylation and stored at −80°C. Samples were freeze dried for ∼56 h prior to hydrolysis and methylation.

Methylation and sulfuric acid hydrolysis were done simultaneously to the sister samples. Hydrolysis of cell walls was done following the method of Saeman et al. (1954) and was optimized to ensure complete hydrolysis of cell walls for watermelon. AIR (5 ± 0.23 mg) was weighed into 2 ml microcentrifuge tubes with screw caps (product no. PFSS 2800 50 20U) (OPS Diagnostics, Lebanon, NJ).

Watermelon samples were treated with 126 μL of 72% sulfuric acid and incubated at room temp for 1 h with intermittent vortexing. Samples were diluted to 1 M with di-water and then placed in an oven at 100°C for 3 h. Samples were removed and cooled to room temperature and brought up to neutral pH using 20% chloroform/dioctyl amine, four rinses are required, followed by a four rinses with chloroform to remove the amine. The internal standard, 10 μL of myo-inositol at 13.56 μg⋅mL^–1^ was added to each sample. Samples were gently mixed and then were dried in a nitrogen-evaporator (Model TM 111, Organomation Associates INC, Berlin, MA) and warm water bath (held at 35°C) and then stored at −20°C until ready for acetylation.

A sister sample was designated for methylation in dimethyl sulfoxide and iodomethane. Samples designated for methylation were pulled from the freeze dryer and 20 μL of methanol were added to dehydrate the sample, then freeze dried for 3 more hours. Samples were solubilized in 200 μL of dimethyl sulfoxide for 18 h. A sodium hydroxide/dimethyl sulfoxide slurry was prepared and 200 μL of the slurry were added to each sample, then were sonicated for 1 h. Iodomethane was added to each sample. After methylation, samples were rinsed three times with di-water and then were dried down under nitrogen in a warm water bath. Methylated samples were hydrolyzed via TFA hydrolysis following the method of Pettolino et al. (2012). Myo-inositol was added to each of the methylated samples (10 μL, 13.51 μg⋅mL^–1^) and samples were dried in a warm water bath (<40°C) under nitrogen.

Hydrolyzed and methylated samples and sugar standards were reduced via the addition of 2 M ammonia and sodium borodeuteride. The reductant was destroyed, and samples were placed in a shallow water bath maintained at about 35°C and evaporated with a stream of nitrogen. Samples were rinsed 2 × 250 μL of 5% (vol/vol) acetic acid/methanol and then 2 × 250 μL methanol to remove borate complexes and stored in methanol overnight at −20°C.

All samples were brought to room temperature prior to acetylation in acetic anhydride. After acetylation samples were packed into small glass vials and 0.1–0.2 g of sodium sulfate was added to remove excess water prior to GC-MS analysis. The dichloromethane phase was transferred into 2 ml vials with PTFE/RS screw caps (part nos. 5182-0715 and 5185-5820, Agilent, Santa Clara CA) Samples were stored at −80°C until ready to run on the GC-MS.

### Partial Methylation of Sugar Standards

Sugar standards of arabinose, glucose, galactose, xylose, mannose, rhamnose, and fucose were converted into partially methylated methyl glycosides following Sassaki et al. (2005) and Wang et al. (2007). In short, 20 mg of standard were weighed separately into 7 mL glass test tubes, weight recorded, and 2 mL of 2% methanolic-hydrochloric acid added. The samples were placed into a water bath (70°C) for 12 h. Samples were removed from the warm bath and 0.15 g sodium bicarbonate (ACS reagent, = 99.7%, cas no. 144-55-8, Sigma Aldrich, St. Louis, MO) was added to neutralize each sample. Samples were filtered with 0.2 μm PTFE filters (13 mm syringe filter, PTFE membrane, cat no. 28145-491, VWR, Radnor, PA) and were dried down in a warm water bath held at 40°C under nitrogen.

The prepared methyl glycosides (e.g., sugar standards) were partially methylated by adding 330 μL dimethylformamide (HPLC, = 99.9%, CAS no. 270547, Sigma Aldrich, St. Louis, MO), 33.2 mg barium oxide (99.99% trace metal basis, CAS no. 554847, Sigma Aldrich) and 1.7 mg barium hydroxide octahydrate (=98%, CAS no. 12230-71-6, Sigma Aldrich). The samples were gently vortexed for 15 s. and 166 μL methyl iodide (grade, Sigma Aldrich) was added. Samples were then placed on an orbital shaker (I 24 Incubator Shaker Series, New Brunswick Scientific, Edison, NJ) at 220 rpm for 4 h. in the dark. Methylene chloride (500 μL) was added and samples were vortexed for 120 s. to extract the partially methylated methyl glycosides. After methylation, samples were rinsed three times with di-water and then were dried down under nitrogen in a warm water bath. The partially methylated samples were hydrolyzed via TFA hydrolysis following the method of Pettolino et al. (2012). Myo-inositol was added to each of the methylated samples 10 μL, 13.51 μg⋅mL^–1^) and samples were dried in a warm water bath (<40°C) under nitrogen.

Sugar standards were reduced via the addition of 2 M ammonia and sodium borodeuteride. Samples were rinsed with acetic acid/methanol and then methanol to remove borate complexes and stored in methanol overnight at −20°C. Samples were dried down with nitrogen and acetylated by adding 400 μL acetic anhydride followed by holding in a 100°C oven for 2.5 h. After acetylation, samples were extracted into 750 mL methylene chloride then packed into small glass vials with PTFE/RS screw caps and 0.1 g sodium sulfate and stored at −80°C until GC-MS analysis.

### Gas Chromatograph-Mass Spectrophotometer and Data Processing

An Agilent 7890A gas chromatography (GC) coupled to a 5975C VL MSD mass spectrometer (MS) (Agilent, Santa Clara, CA) was used for sample analysis. The GC-MS system and injection methods were set up using Agilent 5975c software (library or databases were not) and were adapted from Pettolino et al. (2012). Helium was used as the carrier gas with a flow rate of 1 mL⋅min^–1^. Samples were injected at 1 μL with a split (1/10) injection using an autosampler (inject samples with the temperature at 240°C). Five syringe washes with dichloromethane were preformed between each sample. The oven conditions were set to an initial temperature of 170°C, held for 2 min, and then ramped at 3°C min^–1^ to 260°C. Held for 3 min and the mass selective detector (MSD) transfer line was held at 260°C.

The MS quad was maintained at 106°C and MS source at 230°C, 70 eV electron impact ionization and data were acquired in scan mode from 100 to 350 m/z at 2.14 scans per 1 s and with a solvent delay of 3 min. A BPX 70 column was used, trimmed and purged before starting sample injections. Injector syringe, septa (premium inlet septa, part no. 5183-4757, Agilent, Santa Clara, CA) and inlet liners (liner 4 mm, part no. 5181-3316, Agilent, Santa Clara, CA) were changed from the autosampler to the GC system every 50 injections.

All data were integrated and quantitated via 5975C Data Analysis MSD Chemstation (Agilent, Santa Clara, CA). Alditol acetates (neutral sugars) were identified using relative retention time (rrt) and mass spectra of authentic standards, and each alditol acetate concentration was calculated via comparing samples to a 5 point internal standard curve. Quantitative estimates of each alditol acetate were normalized to the starting AIR mass and are reported in μg⋅mg^–1^ of AIR. The degree of methylation and uronic acid (galacturonic and glucuronic acid) concentrations were also calculated following methods from Trandel et al. (2020b).

Partially methylated alditol acetates (linkage residues) were identified using the partially methylated methyl glycosides generated in this study. The watermelon cell wall chromatograms were compared to the independent methyl glycoside chromatograms and rrts found in Supplementary Table 1 of Pettolino et al., 2012. Some partially methylated glycosides coeluted at the same time. For these, linkage residues were identified using mass spectral data within each independent methyl glycoside and were compared to the mass spectral data from the Complex Carbohydrate Research Center (Georgia, United States) http://www.ccrc.uga.edu/specdb/ms/pmaa/pframe.html. Once the linkages were identified, the glucose and galactose linkages with uronic acid components were calculated using the peak area of *m/z = 119*. All neutral sugar and uronic acid linkages in each watermelon sample were normalized against the internal standard by dividing the peak area of the linkage by the peak area of the internal standard. Then the concentration of each PMAA was estimated from the concentration of the internal standard. All linkages are reported in μg⋅mg^–1^ of watermelon AIR.

### Statistical Analysis

All fruit quality data were subjected to analysis using R 3.5.3 (Revolution Analytics, Mountain View, CA). A one-way ANOVA was run on the incidence of hollow heart (%) between grafting treatments. A two-way ANOVA was run on heart firmness (N), rind firmness (N), soluble solids content, and pH modeled on grafting treatment and hollow heart phenotype. Data were subjected to Bonferroni correction for mean separation (*p* = 0.05).

Compositional data were subjected to analysis using SAS 9.4 (SAS Institute, Cary, NC). A two-way ANOVA was run to determine whether lycopene, arginine, citrulline, total sugars, fructose, glucose, sucrose and mineral contents differ among watermelon from grafting treatment or hollow heart phenotype. Students T-test was done on the significant main effects for mean separation (*p* = 0.05). Tukey’s honest significant difference test was conducted on any significant interaction between graft and hollow heart, *p* = 0.05.

All cell wall polysaccharide data (neutral sugar concentrations, degree of methylation, uronic acid concentration and individual polysaccharide linkages) and total pectin data were analyzed using JMP, Version 15.1 (SAS Institute, Cary, NC). A two-way ANOVA was used to model the effects of graft treatment and hollow heart phenotype on total cell wall material, water soluble, alkali soluble, carbonate soluble, and unextractable pectic fractions, total neutral sugars (nmole⋅mg^–1^), total uronic acids (nmole⋅mg^–1^), each of the 12 neutral sugars, degree of methylation, galacturonic and glucuronic acids, and individual polysaccharide linkages. Where significance was found, a Student’s *T*-test was used for mean separation between the main effects *p* = 0.05. Tukey’s honest significance test was done for mean separation where there was significant interaction of graft × hollow heart (*p* = 0.05).

## Results

### Fruit Quality Attributes

**FIGURE 2:**
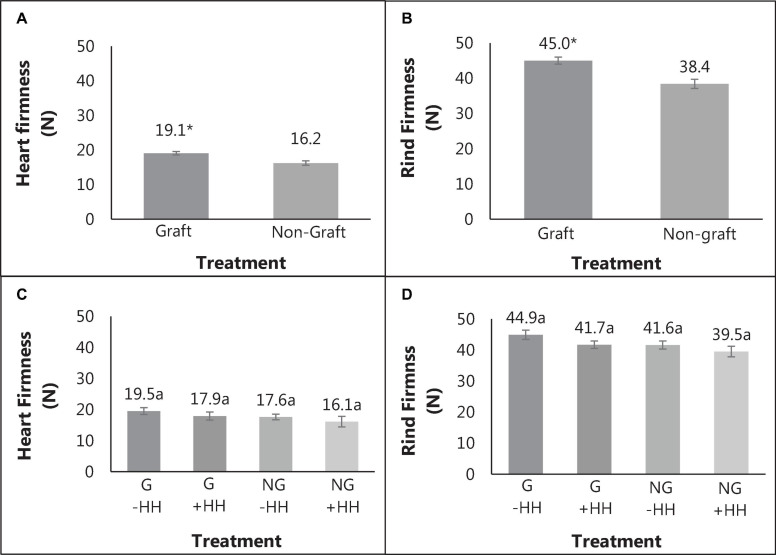
Heart tissue firmness **(A)** and rind firmness **(B)** in fruit from grafted (G) and non-grafted (NG) plants and with (+HH) and without hollow heart (-HH) **(C,D)**, with standard error bars. Mean separation of values from graft and non-graft was done using Bonferroni correction (*p* = 0.05), with differences indicated by *. The significance of graft and HH interactions were determined using Tukey’s honest significant difference effects where different letters indicate significance (*p* = 0.05).

The percent of watermelon fruit with HH was substantially decreased with grafting (19 vs. 53%) while the severity of HH was similar. Fruit weight, soluble solids content and pH were not significantly different with graft or with HH (Table 1). Heart tissue firmness and peel/rind resistance to puncture was higher in fruit from grafted plants compared to fruit from non-grafted plants (Figures 2A,B). Within grafting treatments, fruit with or without HH were similar in flesh firmness and resistance to puncture (Figures 2C,D). Peel/rind firmness was higher in fruit from grafted plants than those from non-grafted (45.1 and 38.4 N). Fruit from grafted plants with and without HH indicated no differences in rind firmness (Figures 2B,D).

**TABLE 1:** Quality measurements in fruit from grafted and non-grafted plants and in fruit with and without hollow heart.

Variable	Soluble solids (°Brix)	Fruit pH
Graft	12.3 ± 1.0	5.4 ± 0.2
Non-graft	12.1 ± 1.5	5.5 ± 0.3
**Hollow heart**		
Graft –HH	12.1 ± 1.6^a^	5.4 ± 0.2^a^
Graft +HH	12.3 ± 1.4^a^	5.5 ± 0.2^a^
Non-graft –HH	11.9 ± 1.0^a^	5.4 ± 0.2^a^
Non-graft +HH	12.1 ± 1.8^a^	5.5 ± 0.2^a^

*Quality measures are reported as LS-means ± standard errors are within the main effects of graft were separated using Students T-test (p = 0.05). Dissimilar means are reported with different letters. Tukey’s honest significance test done for mean separation in the interaction of graft* × *hollow heart (p = 0.05). Dissimilar means are reported with different letters. HH, without hollow heart; +HH, with hollow heart.*

Total lycopene, free arginine and free citrulline contents were similar in tissues regardless of grafting treatment or presence of HH (Table 2). Total lycopene averaged 61.2 ± 0.9 mg⋅kg^–1^, arginine was 1.2 ± 0.2 g⋅kg^–1^, and citrulline was 3.9 ± 0.7 g⋅kg^–1^, respectively. Total sugars, glucose and fructose concentration did not differ with grafting or incidence of HH. Total sucrose content in watermelon flesh was higher than fructose and glucose but did not differ with HH or graft. When expressed as percent of total sugars, %glucose and %fructose averaged 17.5 ± 1.0 and 31.3 ± 0.7, respectively and were not different with treatment (data not reported). The percentage of sucrose differed and %sucrose was higher in fruit with HH (52.2%) compared to fruit without HH (43.4%) but did not differ with grafting.

**TABLE 2:** Phytonutrients, total sugars, sucrose, glucose, and fructose in watermelon fruit from grafted and non-grafted plants and in fruit with or without hollow heart.

Variable	Lycopene (mg⋅kg^–1^)	Arginine (g⋅kg^–1^)	Citrulline (g⋅kg^–1^)	Total sugars (g⋅kg^–1^)	Sucrose (g⋅kg^–1^)	Glucose (g⋅kg^–1^)	Fructose (g⋅kg^–1^)	Sucrose (%)
Graft	63.0 ± 0.5	1.1 ± 0.2	3.4 ± 0.6	94.6 ± 2.3	42.4 ± 3.3	18.7 ± 2.4	33.4 ± 6.6	44.8 ± 7.6
Non-graft	59.7 ± 1.1	1.3 ± 0.2	3.4 ± 0.7	95.8 ± 3.6	44.5 ± 2.5	18.5 ± 2.2	32.4 ± 1.7	46.2 ± 2.5
**Hollow heart**								
No HH	58.9 ± 0.8	1.1 ± 0.2	5.4 ± 0.7	95.2 ± 3.1	43.5 ± 7.3	16.4 ± 2.3	29.7 ± 3.1	44.8 ± 3.3
HH	63.1 ± 0.9	1.3 ± 0.2	3.5 ± 0.6	98.6 ± 2.7	51.8 ± 9.2	18.6 ± 0.9	32.9 ± 2.3	52.2 ± 1.2*

Phytonutrients are reported as LS-means ± SE and means separated within the main effects of graft or HH using Students T-test (p = 0.05). Significant differences within column and variable are indicated by *.

### Fruit Mineral Content

Total nitrogen, magnesium, sulfur, calcium, and iron were similar for fruit from grafted or non-grafted plants or in fruit with and without HH (data not shown). Watermelon fruit tissue had a mean content of 12.5 ± 0.7, 14.4 ± 0.3, 7.5 ± 0.1, 1.1 ± 0.5, 6.5 ± 0.2 g⋅kg^–1^ and 21.4 ± 2.5 mg⋅kg^–1^ total nitrogen, potassium, magnesium, sulfur, calcium and iron, respectively. Other minerals that differed in fruit from grafted or non-grafted plants and with and without HH are reported in Table 3. Phosphorus did not differ with grafting treatment but was highest in fruit with HH (13.1 g⋅kg^–1^) compared to fruit without HH (2.8 g⋅kg^–1^). Fruit with HH (3.5 mg⋅kg^–1^) had higher amounts of boron compared to fruit without HH (12.8 mg⋅kg^–1^). Copper was higher in fruit from grafted plants (7.1 mg⋅kg^–1^) compared to those from non-grafted plants (5.6 mg⋅kg^–1^). Zinc and manganese were higher in fruit from grafted plants at 20.7 and 8.6 ppm, respectively, compared to non-grafted plants (18.2 ad 6.8 mg⋅kg^–1^). Fruit with HH had higher amounts of copper, zinc, and manganese (6.8, 20.6, and 8.1 mg⋅kg^–1^) compared to those with no HH (6.2, 18.1, and 7.1 mg⋅kg^–1^).

**TABLE 3:** Differences in mineral contents in watermelon fruit due to grafting or hollow heart phenotype.

Variable	Phosphorus (g⋅kg^–1^)	Calcium (g⋅kg^–1^)	Copper (mg⋅kg^–1^)	Boron (mg⋅kg^–1^)	Zinc (mg⋅kg^–1^)	Manganese (mg⋅kg^–1^)
Graft	4.6 ± 0.1	6.4 ± 0.3	7.1 ± 0.9*	12.9 ± 1.2	20.7 ± 0.7*	8.6 ± 2.4*
Non-graft	4.1 ± 0.6	6.6 ± 0.2	5.6 ± 1.1	13.2 ± 1.3	18.2 ± 0.9	6.8 ± 1.0
**Hollow heart**						
No HH	2.8 ± 0.2	6.7 ± 0.2	6.2 ± 0.1	12.8 ± 0.3	18.1 ± 3.8	7.1 ± 0.4
HH	3.1 ± 0.2*	6.5 ± 0.2	6.8 ± 0.3*	13.5 ± 0.3*	20.6 ± 1.5*	8.1 ± 0.7*

Minerals are reported as LS-means ± standard errors. Significant differences within column and variable are indicated by *, Students T-test (p = 0.05).

### Total Pectin Content

Total cell wall material, reported as AIR, was calculated as a percentage of fresh watermelon tissue weight and was not different with graft or HH treatments. Sequential fractions are reported as a percentage of that extracted from AIR. The WSF was lowest at 12.82%, while CSF was 25.97% and ASF was highest at 34.58% of TCWM, respectively (Table 4). The CSF was lower in fruit with HH (24.11%) than fruit without HH (27.90%) (*p* < 0.0428). No other differences were found among the pectic fractions. Total neutral sugars and uronic acids of the pectic fractions were not different with graft or HH (Table 5).

**TABLE 4:** Watermelon total cell wall material and sequential fractions (% of total) in relation to grafting and incidence of hollow heart.

		% sequential fraction			
Variable	TCWM	WSF	CSF	ASF	UNX
Graft	0.39 ± 0.02	12.56 ± 0.8	25.77 ± 1.0	34.02 ± 1.0	27.29 ± 1.2
Non-graft	0.36 ± 0.02	13.09 ± 0.9	26.21 ± 1.2	35.13 ± 1.0	25.49 ± 1.3
**Hollow heart**					
No HH	0.37 ± 0.02	12.60 ± 0.9	27.11 ± 1.8*	33.91 ± 1.0	25.78 ± 1.2
Severe HH	0.36 ± 0.02	13.05 ± 0.9	24.79 ± 1.5	35.24 ± 1.0	26.98 ± 1.2

Values represent LS-means ± standard errors. Means are separated within column and variable using Students T-test and significance (p = 0.05) is indicated by *. TCWM, total cell wall material; WSF, water soluble fraction; CSF, carbonate soluble fraction; ASF, alcohol insoluble fraction; UNX, unextractable fraction.

**TABLE 5:** Total neutral sugars and uronic acids among the sequential cell wall fractions for watermelon from differing grafting treatments and incidence of hollow heart.

Variable	WSF	CSF	ASF
**Total neutral sugars (nmole⋅mg**^–^**^1^)**			
Graft	18.36 ± 1.0	8.91 ± 0.2	12.31 ± 0.7
Non-graft	19.17 ± 1.0	8.61 ± 0.2	12.11 ± 0.9
No HH	19.41 ± 1.0	8.54 ± 0.2	12.02 ± 0.9
Severe HH	18.12 ± 1.1	8.98 ± 0.2	12.40 ± 0.9
**Total uronic acids (nmole⋅mg**^–^**^1^)**			
Graft	8.86 ± 0.5	5.08 ± 0.1	6.10 ± 0.3
Non-graft	9.35 ± 0.5	5.18 ± 0.2	6.24 ± 0.3
No HH	9.39 ± 0.5	4.98 ± 0.1	6.12 ± 0.3
Severe HH	8.82 ± 0.5	5.28 ± 0.1	6.22 ± 0.3

*Values represent LS-means ± standard errors. Means are separated within column and variable using Students T-test WSF, water soluble fraction; CSF, carbonate soluble fraction; ASF, alcohol insoluble fraction.*

### Monomeric Composition of Cell Wall Polysaccharides

**FIGURE 3:**
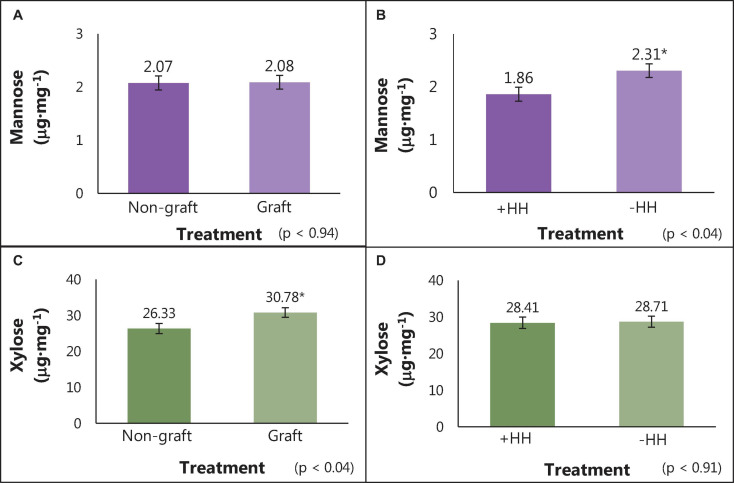
Mannose and xylose content (μg⋅mg^–1^) of cell wall polysaccharides in watermelon fruit from grafted and non-grafted plants **(A,C)** and in fruit with and without HH **(B,D)**. Bars represent means (*n* = 12) ± standard error bars. Student *T*-test was used for mean separation (*p* = 0.05) with significant differences indicated by *.

The average concentration of individual cell wall polysaccharide monomers (alditol acetates) and the degree of methylation (%) for watermelon cell wall polysaccharides are reported in Table 6. No differences were found in meso-erythritol, rhamnose, fucose, arabinose, 2-deoxy-d-glucose, allose, glucose, and galactose concentrations for graft, hollow heart, or the interaction of graft and HH (*p* > 0.05). No 2-deoxy-d-ribose was detected in watermelon cell walls. Fruit with HH were higher in mannose (2.31 μg⋅mg^–1^), compared to fruit without HH (1.85 μg⋅mg^–1^) but no differences were seen with grafting treatment (Figures 3A,B). In contrast, xylose was highest in fruit from grafted plants (30.78 μg⋅mg^–1^) compared to fruit from non-grafted plants (26.33 μg⋅mg^–1^) with no differences in fruit with or without HH (Figures 3C,D). No differences were seen in galacturonic and glucuronic acid concentrations or the degree of methylation (%methylation) with graft or HH.

**TABLE 6:** Average concentration of monomeric building blocks, degree of methylation and galacturonic and glucuronic acids in watermelon cell walls.

Monosaccharide	Concentration (μg⋅mg^–1^ ± SE AIR)
Glucose	78.22 ± 3.26
Galactose	48.94 ± 1.89
Xylose	27.65 ± 1.14
Arabinose	19.72 ± 1.11
Rhamnose	3.26 ± 0.12
Fucose	2.21 ± 0.10
Mannose	2.03 ± 0.10
Ribose	1.15 ± 0.05
Allose	0.48 ± 0.08
2-deoxy-d-Glucose	0.38 ± 0.06
Meso-Erythritol	0.015 ± 0.01
2-deoxy-d-Ribose	−
Galacturonic acid	26.28 ± 2.14
Glucuronic acid	10.15 ± 0.84

**Degree of methylation**	**%**

%Methylation	72.11 ± 5.87

*– = no 2-deoxy-d-ribose detected in watermelon cell wall samples. Values represent LS-means ± standard error, N = 24.*

### Partially Methylated Alditol Acetate Linkage Assembly

**FIGURE 4:**
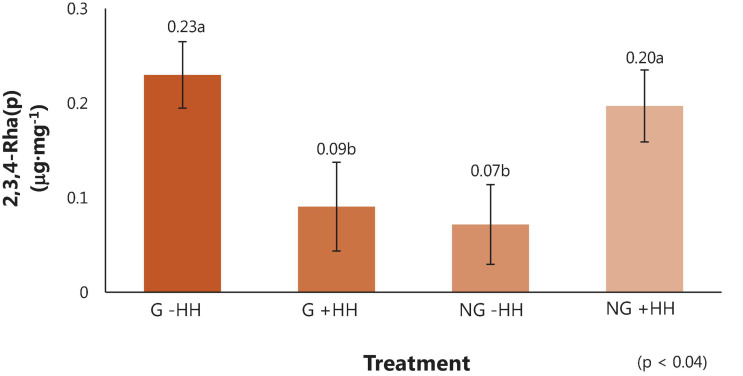
Linkage residue of the partially methylated alditol acetates (μg⋅mg^–1^) ± SE in fruit tissue from watermelon from grafted (G) or non-grafted (NG) plants and with no (-HH) or severe (+HH) hollow heart. Means separated using Tukey’s honest significant difference, with different letters indicating significance (*p* = 0.05).

Comprehensive linkage assembly data are reported as averages in Table 7. From the partially methylated methyl glycosides (e.g., sugar standards), 56 partially methylated alditol acetates were identified with 34 polysaccharide linkage residues found in watermelon cell walls. In watermelon, 4-Glc(p) (37.91 μg⋅mg^–^1), 4-Gal(p) (15.97 μg⋅mg^–1^)), 6-Glc(p) 5.35 μg⋅mg^–1^), 5-Ara(f) (4.72 μg⋅mg^–1^), 3,4-Gal(p) (3.75 μg⋅mg^–1^), t-Ara(f) (2.59 μg⋅mg^–1^), and t-Xyl(p) (2.07 μg⋅mg^–1^) were found to be the linkages in highest abundance on average. The linkage 2,3,4-Rha(p) differed among treatments (Figure 4). However, the relationship between 2,3,4-Rha(p) concentration with grafting and HH phenotype was complex and requires further study to determine whether it has biological significance. No other differences in the content of specific carbohydrate linkages were found in relation to grafting, HH phenotype or their interaction.

**TABLE 7:** Estimated concentrations of watermelon cell wall polysaccharide linkages residues.

Linkage residue	Average concentration (μg⋅mg^–1^ ± SE AIR)	Tentative polysaccharide class
3,4-Gal(p)	3.74 ± 0.43	AG I
3-Ara(f)	0.63 ± 0.07	
4,6-Gal(p)	1.21 ± 0.11	
3-Gal(p)	1.02 ± 0.16	AG II
2-Rha(p)	0.32 ± 0.04	RG I
2,3,4-Rha(p)	0.16 ± 0.05	RG II
2,3,4-Fuc(p)	0.06 ± 0.02	
4-Xyl(p)	0.39 ± 0.07	HX
t-Fuc(p)	0.33 ± 0.05	
2,3-Man(p)	0.25 ± 0.10	HM
2,4-Man(p)	0.46 ± 0.04	
4,6-Man(p)	0.13 ± 0.19	
t-Xyl(p)	2.08 ± 0.29	XG
2-Gal(p)	0.16 ± 0.03	
2,3,4-Xyl(p)	0.20 ± 0.01	
2,4-Gal(p)	0.22 ± 0.05	HG
6-Glc(p)	5.35 ± 0.49	Mixed linked β-glycans
3,4,6-Glc(p)	0.99 ± 0.14	
3-Glc(p)	0.19 ± 0.02	
2-Glc(p)	0.17 ± 0.01	
2,3-Glc(p)	0.06 ± 0.01	
4-Glc(p)	37.91 ± 3.45	Cellulose, XG, and mixed linked β-glycans
4-Gal(p)	15.97 ± 1.65	AG I & II and RG I & II
6-Gal(p)	0.22 ± 0.04	
t-Glc(p)	1.04 ± 0.08	AG I & II, HX, and RG II
2-Ara(f)	0.12 ± 0.03	
5-Ara(f)	4.72 ± 0.43	
t-Ara(f)	2.59 ± 0.21	AG I & II, HM, and XG
t-Gal(p)	0.78 ± 0.08	XG, AG II, and RG I & II
3,4-Fuc(p)	0.32 ± 0.04	RG II and XG
2,3,4-Ara(p)	0.08 ± 0.01	AG and AG protein
2,5-Ara(f)	0.55 ± 0.08	Arabinan
3,4,6-Gal(p)	1.46 ± 0.13	Unknown
2,4,6-Glc(p)	0.18 ± 0.03	
**Uronic acid linkage residues**		
t-GlcA(p)	0.08 ± 0.01	RG II
t-GalA(p)	0.07 ± 0.01	HG, RG I, and II
4-GalA(p)	1.29 ± 0.13	
4-GlcA(p)	2.19 ± 0.19	
3,4-GalA(p)	0.25 ± 0.03	RG I and II
4,6-GalA(p)	0.06 ± 0.01	AG I linked to HG

*Values represent LS-means of partially methylated alditol acetates ± SE. AG, arabinogalactan; RG, rhamnogalacturonan; HX, heteroxylan; HM, heteromannan; XG, xylogalacturonan.*

## Discussion and Conclusion

### Hollow Heart Incidence and Grafting Effects

Factors known to induce HH include heavy rainfall events, temperature fluctuations, pollen viability and pollinator activity during the time of pollination (Fiacchino and Walters, 2003; McGregor and Waters, 2014). Hollow heart is most common in triploid watermelon, where blooms cannot produce viable pollen. Watermelon pollination begins from ∼3 to 6 weeks after transplant and can continue for an additional 6 weeks (Dittmar et al., 2009; Trandel et al., 2020a). Diploid and triploid watermelon flowers open in the morning and flowers typically senesce within a few hours. Triploid pistillate flower stigmas are most receptive to pollen between 15 and 26°C (Lyu et al., 2019) and generally require between 16 and 24 pollinator visits for adequate pollination (Fiacchino and Walters, 2003). In this study, morning temperature fluctuations were not extreme enough to effect pollen viability and pollinator movement during the time of peak pollination (Figure 1A). Only two rainfall events of ∼4 cm occurred and were scattered during production making, rainfall an unlikely contributor to HH defect (Figure 1B).

Grafting “Liberty” watermelon onto an interspecific squash hybrid RS decreased the incidence of HH in watermelon, and increased flesh firmness and peel/rind resistance to puncture. Interspecific hybrid RS have been reported by others to increase watermelon flesh firmness (Soteriou et al., 2017), while other RS such as *Lageneria* or wild watermelon do not seem to have this effect. Rind thickness has been reported to increase in grafted fruit (Yuan et al., 2016) and may contribute to resistance to puncture. Resistance to probe penetration through rind and peel was 7 N higher and flesh firmness was about 3 N higher in fruit from grafted plants compared to those from non-grafted plants, regardless of HH phenotype (Figure 2). Increased rind/peel firmness was found to increase in watermelons grafted to interspecific hybrid rootstocks and was associated with increased fruit flesh firmness (Yuan et al., 2016). Yuan et al. (2016) speculated that increased flesh firmness was due to the increased root size and plant vigor associated with the interspecific hybrid rootstock as mature watermelon fruit in their study also had higher amounts of calcium, magnesium, iron, and zinc.

Watermelon cultivars with lower tissue firmness are generally more susceptible to internal fruit disorders (Trandel et al., 2020a). Increased watermelon firmness has been related to more cells/unit area in the placental tissue (Kano, 1993; Trandel et al., 2021). Trandel et al. (2021) found fruit firmness increased by 1 N in watermelon grafted onto two interspecific hybrid RS of “Carnivor” and “Kazako” compared to non-grafted fruit. Using confocal microscopy to assess watermelon flesh density and its relationship to firmness and HH, cell area decreased with increasing HH severity in fruit when grown without grafting or on “Carnivor” RS. Results were the opposite for fruit from plants grafted to *Laginaria siceraria* RS, and average cell size was largest (Trandel et al., 2021). These results suggest that increased firmness may decrease the tension imposed on the placental tissue, ultimately decreasing middle lamella separation, leading to smaller intracellular air spaces, and less development of HH.

### Sugar and Phytonutrient Composition in Grafted Watermelon With and Without Hollow Heart

Aside from watermelon’s delicious flavor, the fruit is rich in carotenoids and citrulline, providing nutritional and bioactive benefits (Kong et al., 2017). Increased sugars, citrulline, and lycopene in fruit from watermelon grafted on interspecific hybrid rootstocks have been reported in some studies (Davis et al., 2008; Soteriou et al., 2014). These increases may depend on location of the study, environmental conditions, and relative ripeness. Grafting to an interspecific hybrid RS will delay ripening by 5–7 days compared to non-grafted fruit (Fallik and Ilic, 2014). This delay is thought to be from the stress and healing of the graft union, leading to a 3–5 days delay in flower production that is carried through ripening (Kyriacou et al., 2016).

Fruit soluble solids content is the primary indicator of fruit ripeness, and pH is another indicator, with watermelon considered ripe between pH values of 5.0–5.5 (Corey and Schlimme, 1988; Soteriou et al., 2014; Kyriacou et al., 2016). Watermelon fruit in our study were fully ripe, as indicated by pH values near 5.5 for grafted and non-grafted samples (Table 1). A soluble solids content greater than 8% is commonly used as a benchmark for ripe watermelon (Kyriacou et al., 2018), and was about 12% in all fruit in this experiment. In watermelon, SSC is primarily from the soluble sugars sucrose, glucose, and fructose (Fall et al., 2019). Total sugars, glucose, sucrose, and fructose did not differ with HH or graft (Table 2). When sucrose was calculated as percent of total sugars, no differences were seen with grafting although fruit with HH were found to be higher in % sucrose than fruit without HH (Table 2). During fruit ripening, sucrose is the main carbohydrate accumulating as glucose and fructose decrease (Kyriacou et al., 2018). Sucrose accumulation occurs during ripening via sucrose phosphate synthase and sucrose synthase activities and decreased acid invertase activity (Yativ et al., 2010; Kyriacou et al., 2018). The increased proportion of sucrose in HH fruit without a concomitant increase in overall sugars indicates a directed enzyme activity, possibly in response to HH development.

A full red coloration in watermelon is an indicator of quality. The red pigment is from lycopene and color development begins in the locular regions shortly after cell division ends and extends progressively to 43–45 days after pollination (Kyriacou et al., 2018). Lycopene content has been reported to increase, decrease, or remain unchanged in watermelon grafted to interspecific rootstocks, when care has been taken to select fruit of similar ripeness to those from non-grafted plants (Kong et al., 2017). Results from this study suggest the interspecific hybrid RS “Carnivor” did not up or down regulate lycopene content and HH did not compromise phytonutrient quality (Table 2).

Citrulline is a potent osmolyte and radical scavenger against drought/salt stress in plants (Akashi et al., 2001) as well as an intermediate in the human nitric oxide system. In studies done with 14 and 56 watermelon cultivars, no correlation was found with watermelon type (e.g., open-pollinated and hybrid cultivars) and citrulline content (Rimando and Perkins-Veazie, 2005; Kyriacou et al., 2018). Results have been inconclusive regarding citrulline content and watermelon grafting as some rootstock-scion combinations have been shown to increase citrulline content (Davis et al., 2008) or have no effect with grafting (Aslam et al., 2020). In our study, citrulline did not change substantially either with grafting or hollow heart (Table 2).

### Graft and Hollow Heart Effects on Fruit Minerals

When watermelon is grafted with a compatible rootstock and a vigorous root system is adopted, plants absorb and shuttle mineral nutrients more efficiently than non-grafted watermelon (Ruiz et al., 1997; Martinez-Ballesta et al., 2010). Likewise, the cell wall can also play a role in mineral shuttling and is responsible for the movement of metal cations into cellular organelles (Yang et al., 2018). Due to increased water and nutrient uptake from watermelon grafting, the cell wall may readily transport copper, manganese and zinc through the cell wall into storage organelles (Ruiz et al., 1997; Martinez-Ballesta et al., 2010), and may have accounted for the significant increase of these minerals in fruit from grafted plants in our study (Table 3).

Fruit with and without HH differed in mineral content (Table 3). Phosphorus aids plant growth and its distribution is affected by sink to source relationships. Phosphorus content in plant tissues can influence fruit quality attributes, as found for increased soluble solids content in strawberries (Cao et al., 2015). Strawberry fruit are a strong sink organs and fruit with higher P have enhanced sugar transport from leaf to fruit (Cao et al., 2015). Higher P content found in watermelon with HH may indicate high sugar enzyme activity suggesting that increases in P increase sucrose synthase activity.

Boron is essential throughout the plant and is responsible for boron-bridging and dimerization of RG II. Boron generates a covalent bridge between pectin molecules and decreases cell wall porosity by modifying biochemical properties of the cell wall (Chormova and Fry, 2015). While the major sites of mineral accumulation for copper, manganese and zinc are in the chloroplast, vacuole and cytoplasm, the cell wall controls heavy metal uptake and storage (Printz et al., 2016). It is possible that the increased boron, zinc and manganese in fruit with HH may be from storage in fruit cells or a change in metal transport mechanisms in the cell wall (Printz et al., 2016). Increased minerals found in fruit from grafted plants might result indirectly from increased water and nutrient uptake from the roots (Table 3).

### Total Pectin Content and Fruit Textural Characteristics

Watermelon tissue firmness is attributed to the strength and intercellular connections of the primary cell wall (Brummell, 2006). Pectin is the most abundant polymer in the middle lamella and regulates intercellular adhesion (Paniagua et al., 2017). The solubilization of pectin varies greatly and fruit with a crisp texture, like watermelon, have a low degree of pectin depolymerization and solubilization when ripe (Brummell, 2006; Soteriou et al., 2017).

Despite the significant increase in heart tissue firmness (Figure 2A), no differences were seen in total cell wall material or sequential pectic fractions (%) with grafting treatment (Table 4). Soteriou et al. (2017) speculated that increased fruit tissue firmness could be elicited by an increase in cytokinin production from the grafting rootstock. Rootstock grafts promote plant growth via synthesis and translocation of cytokinin and gibberellin. Recently, an upregulation of cytokinin was found in watermelon grafted to squash RS, promoting the movement of this hormone from the RS to the scion and subsequent fruit (Liu et al., 2016). Cytokinin is generally synthesized in the roots and mediates local and long-distant signaling that influences fruit qualities by increasing cell division (Srivastava and Handa, 2005). Increases in cell division are related to an increased number of cells/unit area and may be associated with textural changes (Kano, 1993). Grafting onto interspecific hybrid RS has been found to increase root vigor and root dry weight 14 d after pollination compared to non-graft controls (Yuan et al., 2016). This vigor may further promote the differences in fruit quality from increased nutrient shuttling and/or changes in cytokinin response between grafted and non-grafted watermelon in this study.

The %WSF was lowest compared to the %CSF and %ASF, which is comparable to what Soteriou et al. (2017) found in fruit grafted to interspecific hybrid rootstocks and non-grafted watermelon. Similarly, in other crops like strawberry and apple, %WSF was lowest compared to all other fractions (Yu et al., 1996; Ng et al., 2013), while in blueberry all sequential fractions were similar (Lin et al., 2019). Water soluble fractions yield pectins in the plant cell wall that are freely bound and soluble in the apoplast (Paniagua et al., 2014). Consistent with our findings, Soteriou et al. (2017) found no differences in the %WSF in fruit from homeografted, non-grafted and heterografted plants, suggesting a limited association between WSF and tissue firmness. Total neutral sugars were highest in the WSF at 18.76 nmole⋅mg^–1^ compared to all other pectic fractions and neutral sugars were higher compared to total uronic acids (Table 5). Polysaccharides such as XG and RG I and II have neutral sugar side chains. Increases in total neutral sugars in WSF may be due to the pectic sidechains solubilizing in the apoplast of intact cell walls (Redgwell et al., 1992).

The %CSF was found to differ only in watermelons with the HH disorder (Table 4) and was higher in fruit without HH (27.1%) compared to those with HH (24.8%). Carbonate soluble fractions are enriched in de-esterified pectin covalently bound to the cell wall (Paniagua et al., 2014), HG (Mohnen, 2008; Paniagua et al., 2014; Soteriou et al., 2017), and RG I (Paniagua et al., 2014). Fruit with HH may have lower enrichments of RG I and HG. Loss of arabinan and galactan side chains from RG I may further contribute to increased solubilization of CSF in fruit without HH (Zywinska et al., 2005). Carbonate soluble fractions extracted with calcium or sodium carbonate have previously been found to have higher total neutral sugar concentrations compared to total uronic acids. In this study, neutral sugars from CSF were 8.76 nmole⋅mg^–1^ compared to 5.15 nmole⋅mg^–1^ total uronic acids (Table 5). These results suggest CSF from grafted watermelon may have high amounts of RG I (Paniagua et al., 2014; Soteriou et al., 2017).

Alkali soluble fractions are derived from matrix glycans tightly attached to the cell wall via hydrogen bonds and are generally the hardest to solubilize (Brummell and Harpster, 2001). These fractions are also considered to have a low degree of methylesterification (Gawkowska et al., 2019) and high polyuronide (uronic acid) concentrations (Soteriou et al., 2017). In this experiment, ASF were highest compared to all other pectic fractions but had no discernable treatment differences. The ASF also had higher total neutral sugars (12.21 nmole⋅mg^–1^) compared to total uronic acids (6.17 nmole⋅mg^–1^) (Tables 4, 5). Arabinose and galactose were found in high amounts in ASF from apple. Similarly, these neutral sugars are found in high concentrations in watermelon (Brummell, 2006; Soteriou et al., 2017) accounting for higher total neutral sugars compared to uronic acids.

### Role of Neutral Sugar Composition in Watermelon Cell Wall Polysaccharides

Neutral sugars and galacturonic and glucuronic acids are the monomeric building blocks forming complex polysaccharide networks. The net change or loss of specific monosaccharides have been linked to fruit ripening characteristics in strawberry, beet, tomato, stonefruit, melon, starfruit, and watermelon (Brummell, 2006; Karakurt et al., 2008; Petkowicz et al., 2016; Broxterman and Schols, 2018).

In this experiment, 12 neutral sugars were analyzed, and average concentrations are reported in Table 6. Previous research on watermelon focused on the mol% of only the major monosaccharide building blocks (Yu and Mort, 1996; Brummell, 2006). Karakurt et al. (2008) found 95% of the watermelon matrix glycans consisted of 40% glucose, 30% xylose, 15% arabinose, and 14% galactose. Likewise, monosaccharides in highest content in this study were glucose (78.22 μg⋅mg^–1^), galactose (48.49 μg⋅mg^–1^), xylose (27.65 μg⋅mg^–1^) and arabinose (19.76 μg⋅mg^–1^), respectively.

Among the major monosaccharides identified in watermelon, galactose is a structural component of the backbone in pectin and is a major constituent in arabinogalacto-proteins and RG I and II (Jimenez et al., 2001; Kruger et al., 2008; Pogosyn et al., 2013). High concentrations of galactose are expected in watermelon, as this sugar is present in many pectic polysaccharides. Glucose increases in the cell wall as plant and fruit parts ripen (Jimenez et al., 2001), and is a monomeric building block in cellulose, hemicellulose and RG (Pettolino et al., 2012; Chormova and Fry, 2015). Research on olive fruit suggests that increases in glucose are related to increases in RG II (Jimenez et al., 2001). Xylose is a major monosaccharide in the formation of XG, encompassing about 10% of plant cell wall pectins (Kruger et al., 2008). Xylose also plays a role in RG II side branches (Brummell, 2006; Karakurt et al., 2008; Kruger et al., 2008). Arabinose is known to be a pectic sugar primarily and is dominant in building type I and II arabinogalactans (AG), which attach to RG I and XG (Pogosyn et al., 2013; Yang et al., 2018).

Higher amounts of galacturonic acid (GalA) (26.28 μg⋅mg^–1^) were found compared to glucuronic acid (10.15) (Table 6). Coenen et al. (2007) proposed a model for the pectic backbone that is made up of alternating HG and RG I with xylose as side chains. Coenen et al. (2007) found that HG was built of 81-117 GalA residues, while RG I was built of ∼20 GalA residues (Amos and Mohnen, 2019). Due to several polysaccharide backbones being built of GalA, high amounts were expected in watermelon cell walls. Glucuronic acid (GlcA) is expected to be found in lower amounts as it is found only in xylans (made up of 12 GlcA residues), AG backbones (1–8 GlcA residues) and RG II side chains (8–27 GlcA residues) (Chormova and Fry, 2015; Leivas et al., 2015; Amos and Mohnen, 2019).

### Differences in Neutral Sugars With Grafting or Hollow Heart Defect

No 2-deoxy-d-ribose was detected and monosaccharide concentrations of meso-erythritol, rhamnose, fucose, arabinose, 2-deoxy-d-glucose, allose, glucose, and galactose in fruit were similar with grafting and HH. However, differences were seen in mannose concentration; fruit with HH (3 μg⋅mg^–1^) had higher amounts compared to fruit without HH (2.31 μg⋅mg^–1^), while no differences were seen in grafting treatments (Figures 3A,B). Mannose is an important neutral sugar in both pectic and hemicellulosic polysaccharides. A key pectic polysaccharide, RG I, is highly branched and composed of the neutral sugars glucose, fucose, and mannose (Voragen et al., 2009; Erdmann do Nascimento et al., 2017). Hemicelluloses such as heteromannan are composed of β-1,4-mannose residues and thus have mannose present in the polysaccharide structure (Hernandez-Gomez et al., 2015). The increase of mannose in fruit with HH suggest either a change in RG I sidechain configuration or an increase of heteromannan.

Xylose concentration was lower in fruit from non-grafted plants (26.33 μg⋅mg^–1^) compared to those from grafted plants (30.28 μg⋅mg^–1^). Xylose is a critical monosaccharide that encompasses pectic polysaccharides such as the backbone of XG and side branches of RG II (Brummell, 2006; Karakurt et al., 2008; Kruger et al., 2008). Xylose also plays a dominant role in heteroxylan, a hemicellulose composed of β-1,4-xylose residues (Hernandez-Gomez et al., 2015). The lower levels of xylose found in fruit from non-grafted plants imply that less XG or heteroxylan is present.

The degree of methylation (% methylation) has been associated with the rate of cell wall degradation in that higher % methylation is related to increased cell wall disassembly (Krall and McFeeters, 1998; Lurie et al., 2003). Pectin can be demethylated and depolymerized by both enzymatic and non-enzymatic reactions in fruit and vegetables (Krall and McFeeters, 1998). Extensive depolymerization of pectin and removal of neutral sugar side chains happens during fruit ripening (Brummell and Harpster, 2001). Although fruit from grafted plants had increased tissue firmness, no significant differences in the degree of methylation were found relative to watermelon grafting treatment or HH phenotype. In this experiment, the % methylation was highly variable among individual watermelon, which may have influenced the ability to observe differences associated with grafting treatments and/or HH incidence.

### Linkage Assembly of Cell Wall Constituents

Methylation analysis offers a means to elucidate polysaccharide structure and investigate cell wall architecture (Pettolino et al., 2012). Pectic polysaccharides participate in crosslinking interactions as linkage residues are added to terminal ends of polysaccharide chains to synthesize pectic constituents or to connect pectin with other cell wall networks (Amos and Mohnen, 2019). An advantage of determining monosaccharide composition via linkage assembly is the potential to identify the relative proportion of different polysaccharides in the cell walls from one single analysis (Pettolino et al., 2012). In our study, several new linkage residues were found in watermelon cell walls; some of the novel linkages identified include 3,4,6-Gal(p) and 2,4,6-Glc(p).

Linkage residues found in highest concentration in these “Liberty” watermelon were 4-Glc(p), 4-Gal(p), 6-Glc(p), 5-Ara(f), 3,4-Gal(p), t-Ara(f), and t-Xyl(p), respectively (Table 7). There was a significant interaction of graft by HH occurred for the linkage residue 2,3,4-Rha(p), which was lowest in fruit from grafted plants with HH and in fruit from non-grafted plants without HH (Figure 4). The relationship between 2,3,4-Rha(p) and HH phenotype is complex, and further research is needed to understand whether there is any biological significance in the different contents of this linkage.

Galactose linkages are known to play a dominant role in building specific polysaccharides such as AG I and II (Tan et al., 2013; Yamassaki et al., 2018) and HG. In this experiment, 4-Gal(p) and 3,4-Gal(p) were dominant linkages. Arbinogalactan I requires 4-Gal(p), 3,4-Gal(p), 4,6-Gal(p), and t-Ara(f) to build the polysaccharide (Pettolino et al., 2012; Tan et al., 2013; Leivas et al., 2015), and all of these linkages were identified in our study. The high amounts of both 4-Gal(p) and 3,4-Gal(p) support the conclusion by Redgwell et al. (1997) that AG I is a dominant polysaccharide in watermelon fruit cell walls. While for AG II, 3-Gal(p), 6-Gal(p), 2-Ara(f), and 3,4,6-Gal(p) are required, and all linkages were found in watermelon suggesting the presence of AG II. Arabinan, a hemicellulose, is composed of 5-Ara(f), 2,5-Ara(f), 3,5-Ara(f), and t-Ara(f) linkages (Durado et al., 2006; Pettolino et al., 2012). All of these arabinose linkages except 3,5-Ara(f) were found in this experiment and indicate that arabinan is another dominant hemicellulose in watermelon cell walls.

The highly abundant glucose linkages, like 4-Glc(p) and 6-Glc(p), are commonly derived from cellulose, HM or XG (Pettolino et al., 2012). The linkage residue 4,6-Glc(p) found in HM was not identified in this experiment; however, linkages of t-Gal(p), 4,6-Man(p), and 4-Glc(p) were present and indicate HM as another notable polysaccharide in watermelon. The linkage residue of 4-Xyl(p) was another dominant linkage in this experiment, and this linkage is quite common in heteroxylans, a type of hemicellulose (Pettolino et al., 2012; Kang et al., 2018). Heteroxylan is composed of additional linkage residues such as 2,3,4-Xyl(p), t-Ara(f), and t-GlcA in the polysaccharide structure, and all of these linkages were found watermelon. In Arabidopsis and dried distiller grains, the linkage t-Xyl(p) was found to make up hemicelluloses (Kang et al., 2018) and XG (Pettolino et al., 2012). Several XG linkages were identified in this experiment including 4-Glc(p), 2-Xyl(p) 2-Gal(p), and t-Xyl(p), suggesting the presence of XG in watermelon. It is likely that some of the 4-Glc(p) is associated with XG while the majority of the 4-Glc(p) could be attributed to cellulose. The relatively high abundance of 6-Glc(p) along with detection of several other glucose linkages also signifies the presence of mixed linkage β-glycans (Coenen et al., 2006; Pettolino et al., 2012).

### Cell Wall Analysis and Fruit Quality

Interesting and unexpected trends were found in watermelon cell walls in this study. Total pectin and cell wall composition data could not be linked directly to differences in fruit textural characteristics associated with grafting, suggesting other factors may be at play. In apples, fruit textural characteristics were linked to the cell wall by determining the degree of polymerization, enzymatic activity [endo-polygalacturonase (PG) and pectin methylesterase (PME)], tissue microstructure/middle lamella separation and molecular weight of pectin (Ng et al., 2013). Larger pectin molecules were associated with increased apple tissue firmness and pectin immunolabeling gave insight on cell adhesion, cell-to-cell loosening and middle lamella separation (Ng et al., 2013; Pose et al., 2015). Immunolabeling also indicated the type of HG within the middle lamella (Ng et al., 2013). These approaches may be useful in elucidating a cell wall-fruit texture connection in watermelon.

The degree of polymerization is similar to molecular weight analysis and can give insight on the number of residues within a polysaccharide chain (Pose et al., 2015). The degree of polymerization decreased as fruit firmness decreased in strawberry (Pose et al., 2015). Furthermore, enzymes such as PG are thought to depolymerize HG within stretches of unesterified galacturonic acid residues that are created by PME (Brummell and Harpster, 2001; Ng et al., 2013). In apple, increases in PME were related to decreases in highly esterified HG, while increases in PG were found to depolymerize pectin in the cell wall leading to softer fruit (Ng et al., 2013). Enzyme activity of PG and PME in watermelon following ethylene was not found to increase despite tissue softening (Brummell, 2006; Karakurt and Huber, 2009). However, assessing the degree of polymerization/molecular weight has not been done on grafted and non-grafted watermelon and may be useful in understanding fruit textural differences that are not associated with composition alone.

Rootstocks are known to have wide-ranging effects on the regulation of the scion and developing fruit. Watermelon grafted to squash rootstocks were found to differentially express up to 3,485 genes associated with primary and secondary metabolism (Liu et al., 2016; Soteriou et al., 2017). Of these genes, 20 to 47 microRNAs were significantly different in watermelon grafted onto bottle gourd compared to those grafted to interspecific hybrid RS (Liu et al., 2016). Moreover, more genes were differentially expressed in the watermelon grafted to interspecific hybrid squash (36 genes encoding pentatricopeptide repeat proteins and 13 WD40 repeating-containing proteins) compared to the bottle gourd RS (Liu et al., 2016). By analyzing microRNA in grafted and non-grafted plants, researchers may gain better insight as to the role of the rootstock and differences in textural properties between fruit from grafted or non-grafted plants.

## Conclusion

Hollow heart is a serious internal defect found predominantly in triploid watermelon types and cannot be visually distinguished from normal fruit unless cut. In this study, HH was induced on demand by limiting pollen through reducing pollenizer plants in the field to 8% of total watermelon plants. Grafting a HH susceptible triploid cultivar to an interspecific hybrid rootstock increased fruit tissue firmness and decreased HH incidence. Although the presence of HH can cause load rejection, HH did not compromise fruit quality (e.g., firmness, pH, °Brix and total sugar) or phytonutrient content in this study. Additionally, cell wall polysaccharide composition was not related to increased firmness in fruit from grafted plants. Total pectin from CSF was lowest in fruit with HH suggesting these fractions may be enriched in HG. Thirty-four cell wall linkage residues were identified in watermelon for the first time, and arabinan and AG I were found to be notable polysaccharides in watermelon cell walls. Since few differences were found with grafting and fruit textural characteristics, research on tissue microstructure, pectin immunolabeling, enzymatic activity, and degree of polymerization may help delineate the relationship of cell wall polysaccharides to fruit quality and rootstock influences on scion fruit firmness.

## Data Availability Statement

All datasets generated for this study are included in the article/supplementary material, further inquiries can be directed to the corresponding author/s.

## Author Contributions

MT conceived the experimental ideas, performed experiments, and summarized the data. MT, PP-V, and SJ wrote the final manuscript. JS provided information on how to induce hollow heart. JS and CG provided plant production advice. All authors contributed to the article and approved the submitted version.

## Conflict of Interest

The authors declare that the research was conducted in the absence of any commercial or financial relationships that could be construed as a potential conflict of interest.

## Abbreviations

AIR: alcohol insoluble residue
AG I: type I arabinogalactan
AG II: type II arabinogalactan
Ara: arabinose
ASF: alkali soluble fraction
CSF: carbonate soluble fraction
Gal: galactose
GalA: galacturonic acid
Glc: glucose
GlcA: glucuronic acid
HH: hollow heart
-HH: without hollow heart
+HH: with hollow heart
HG: homogalacturonan
HM: heteromannan
HX: heteroxylan
Man: mannose
N: newton
PG: polygalacturonase
PMAA: partially methylated alditol acetates
PME: pectin methylesterase
RCBD: randomized complete block design
Rha: rhamnose
RG I: rhamnogalacturonan I
RG II: rhamnogalacturonan II
RS: rootstock
rt: retention time
rrt: relative retention time
SSC: soluble solids content
TCWM: total cell wall material
WSF: water soluble fraction
XG: xylogalacturonan
Xyl: xylose
UNX: unextractable fraction.
